# Supplementary material for: Visualization of the Serratia Type VI Secretion System Reveals Unprovoked Attacks and Dynamic Assembly
Source: Cell Rep. 2015 Sep 17;12(12):2131–42. doi: 10.1016/j.celrep.2015.08.053 (PMC4594159; doi:10.1016/j.celrep.2015.08.053)
Supplement: Document S1. Figures S1 and S2, and Table S1 [file mmc1.pdf]

Cell Reports

Supplemental Information

Visualization of the *Serratia* Type VI Secretion System Reveals Unprovoked Attacks and Dynamic Assembly

Amy J. Gerc, Andreas Diepold, Katharina Trunk, Michael Porter, Colin Rickman, Judith P. Armitage, Nicola R. Stanley-Wall, and Sarah J. Coulthurst

Figure S1

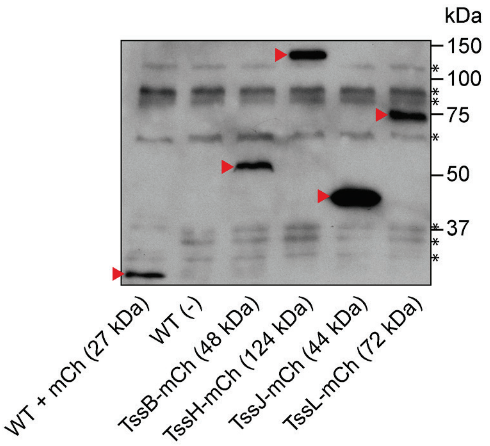

Figure S1. Confirmation of the integrity of chromosomally-encoded fusion proteins of Type VI secretion system components with mCherry, related to Figure 1.

Total cellular fractions of wild type *Serratia marcescens* Db10 (WT) and derivatives expressing fusions of mCherry to the C-terminus of TssB (TssB-mCh), TssH (TssH-mCh), TssJ (TssJ-mCh) or TssL (TssL-mCh) were subjected to immunoblotting using anti-mCherry antisera. A positive control is provided by the WT expressing mCherry from a plasmid (WT + mCh). The predicted molecular weight of each protein is given in parentheses. The mCherry fusion proteins are highlighted with red arrowheads and the positions of non-specific bands are indicated by asterisks on the right hand side.

Figure S2

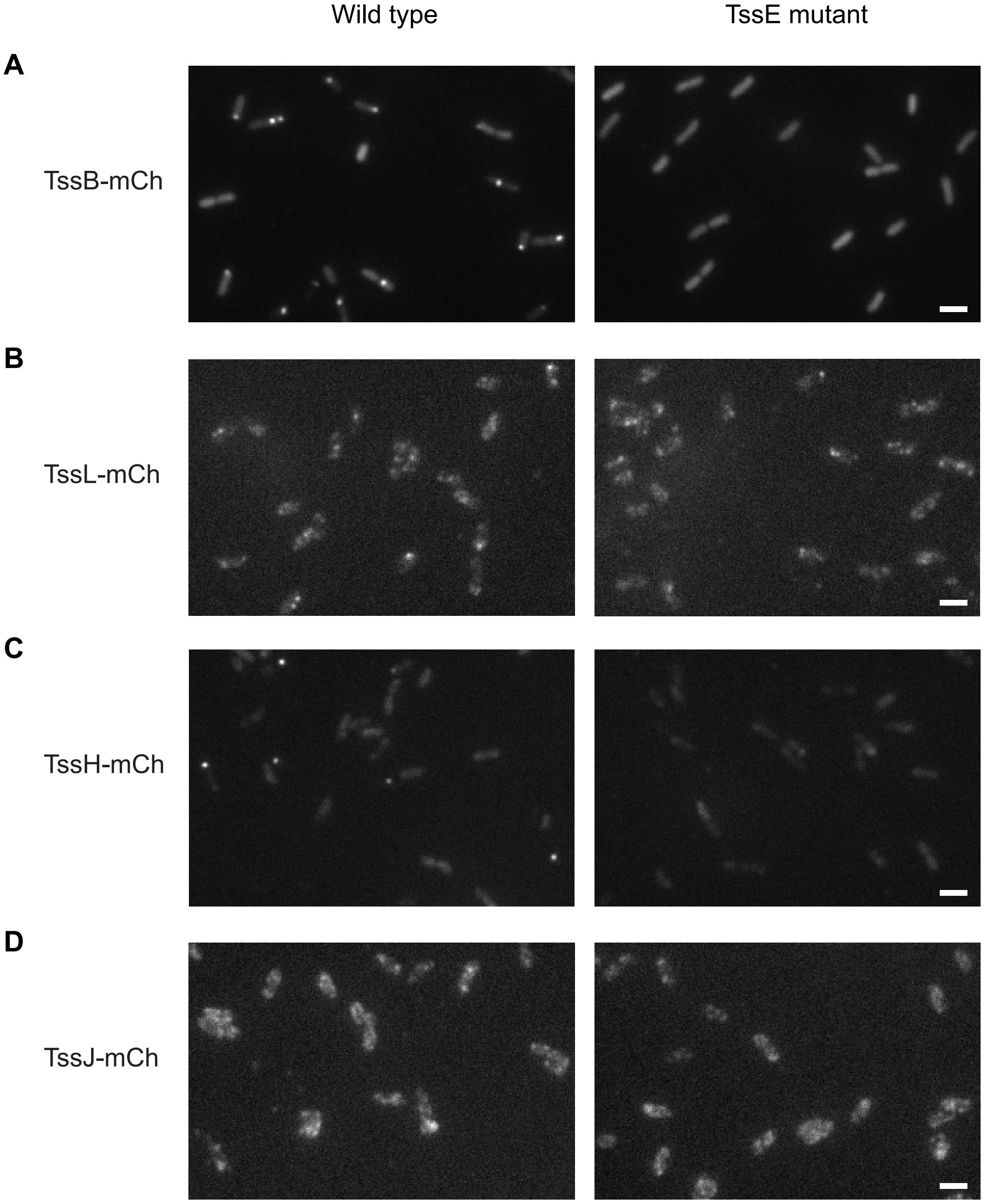

Figure S2. Localisation of TssB, TssH, TssJ and TssL in the absence of the essential baseplate protein TssE, related to Figure 4.
Representative fluorescence images of cells of *Serratia marcescens* Db10 expressing chromosomally-encoded fusion proteins TssB-mCh (A), TssL-mCh (B), TssH-mCh (C) or TssJ-mCh (D), in either a wild type (left) or Δ*tssE* mutant (right) background; scale bar, 2 μm.

Legends for Supplemental Movies

Movie S1. Fluorescence time course of TssB-mCherry during microcolony development, related to Figure 2.
Images were acquired at 10 min intervals for a total period of 6 hours, for the field of view containing the cells shown in Fig. 2. Left panel, DIC images; middle panel, corresponding fluorescence images (mCherry channel); right panel, false-coloured merge of DIC images (greyscale) and fluorescence images (mCherry, red).

Movie S2. Fluorescence time course of TssB-mCherry, related to Figure 4E.
Images were acquired at 10 s intervals for 4 minutes.

Movie S3. Fluorescence time course of TssH-mCherry, related to Figure 4F.
Images were acquired at 10 s intervals for 4 minutes.

Table S1. Bacterial strains used in this study, related to Experimental Procedures

Strain	Description	Source or Reference
S. marcescens Db10	Wild type strain	(Flyg et al., 1980; Iguchi et al., 2014)
SJC3	Db10 Δ*tssH* (ΔSMDB11_2274)	(Murdoch et al., 2011)
SJC11	Db10 Δ*tssE* (ΔSMDB11_2271)	(Murdoch et al., 2011)
SAN157	Db10 *tssJ-mCherry* (TssJ-mCh translational fusion; TssJ, SMDB11_2252)	This study
SAN159	Db10 *tssH-mCherry* (TssH-mCh translational fusion; TssH, SMDB11_2274)	This study
SAN162	Db10 *tssL-mCherry* (TssL-mCh translational fusion; TssL, SMDB11_2254)	This study
SAN163	Db10 *tssB-mCherry* (TssB-mCh translational fusion; TssB, SMDB11_2258)	This study
SAN199	Db10 *tssB-mCherry,* Δ*lacZ*::P_{T5}-*gfpmut2-kan*^{R} (TssB-mCh translational fusion and cytoplasmic GFP; LacZ, SMDB11_2462)	This study
SAN207	Db10 *tssB-gfpmut2*; TssB-GFP translational fusion	This study
SAN208	Db10 *tssB-gfpmut2, tssJ-mCherry* (dual reporter fusion strain)	This study
SAN209	Db10 *tssB-gfpmut2, tssH-mCherry* (dual reporter fusion strain)	This study
SAN210	Db10 *tssB-gfpmut2, tssL-mCherry* (dual reporter fusion strain)	This study
KT24	Db10 ΔT6SS (ΔSMDB11_2244-SMDB11_2281), Sm-resistant	(English et al., 2012)
KT153	Db10 *tssJ-mCherry*, Δ*tssE*	This study
KT154	Db10 *tssH-mCherry*, Δ*tssE*	This study
KT155	Db10 *tssL-mCherry*, Δ*tssE*	This study
KT156	Db10 *tssB-mCherry*, , Δ*tssE*	This study
S. marcescens ATCC274	Wild type strain	Lab stock
SJC44	ATCC274 Δ*tssE*	This study
KT81	Sm-resistant derivative of ATCC274	This study
KT82	Sm-resistant derivative of SJC44	This study
P. fluorescens KT02	Sm-resistant derivative of *Pseudomonas fluorescens* 55	(Murdoch et al., 2011)

Supplemental Reference

Flyg, C., Kenne, K., and Boman, H.G. (1980). Insect pathogenic properties of *Serratia marcescens*: phage-resistant mutants with a decreased resistance to *Cecropia* immunity and a decreased virulence to *Drosophila*. J Gen Microbiol *120*, 173-181.
