# Supplementary figures and images for: Visualization of the Serratia Type VI Secretion System Reveals Unprovoked Attacks and Dynamic Assembly
Source: Cell Rep. 2015 Sep 17;12(12):2131–42. doi: 10.1016/j.celrep.2015.08.053 (PMC4594159; doi:10.1016/j.celrep.2015.08.053)

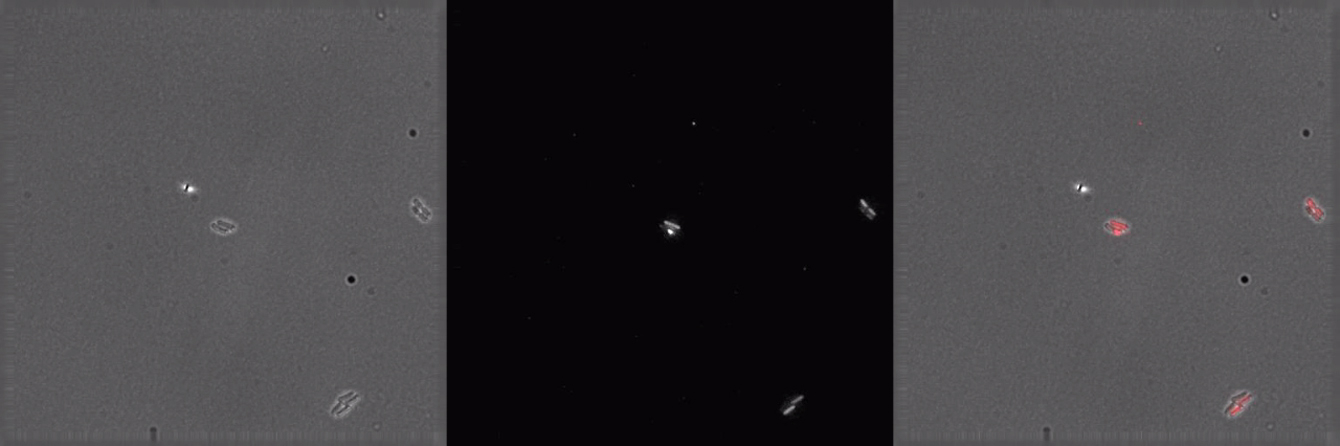

Supplement: Movie S1. Fluorescence Time Course of TssB-mCherry during Microcolony Development, Related to Figure 2 — Images were acquired at 10 min intervals for a total period of 6 hours, for the field of view containing the cells shown in Fig. 2. Left panel, DIC images; middle panel, corresponding fluorescence images (mCherry channel); right panel, false-coloured merge of DIC images (greyscale) and fluorescence images (mCherry, red). [file mmc2.jpg]

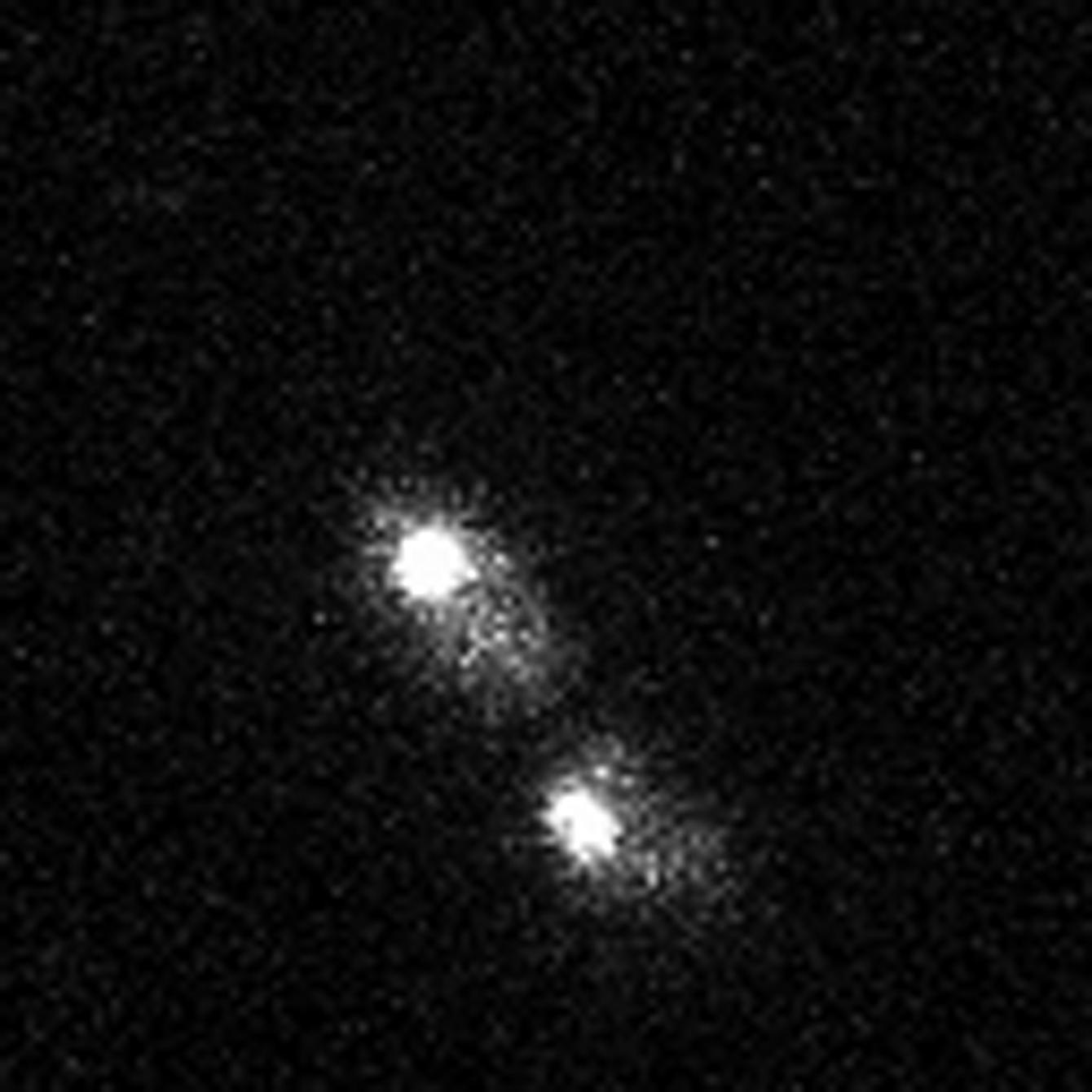

Supplement: Movie S2. Fluorescence Time Course of TssB-mCherry, Related to Figure 4E — Images were acquired at 10 s intervals for 4 minutes. [file mmc3.jpg]

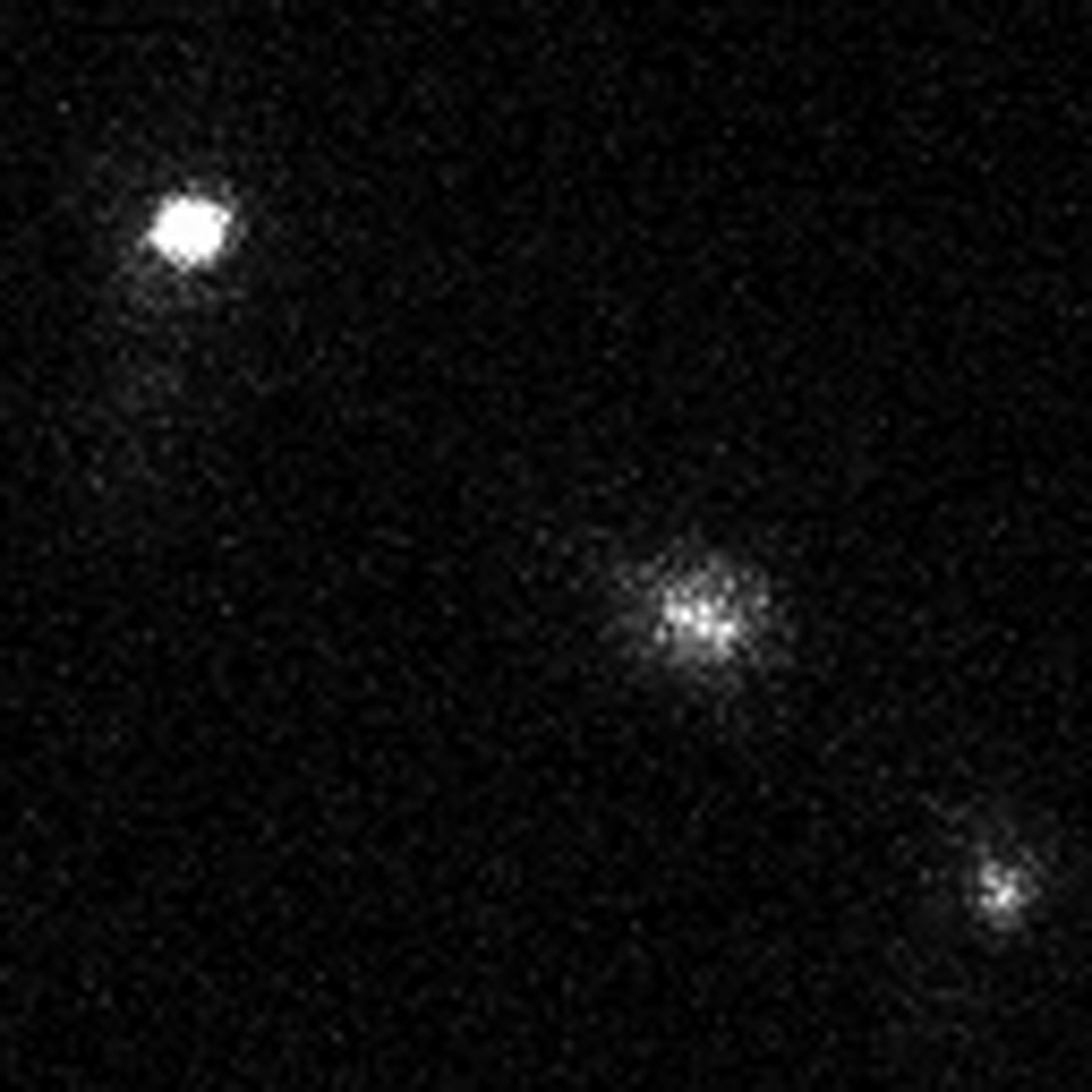

Supplement: Movie S3. Fluorescence Time Course of TssH-mCherry, Related to Figure 4F — Images were acquired at 10 s intervals for 4 minutes. [file mmc4.jpg]
