# Supplementary material for: Visualization of the Serratia Type VI Secretion System Reveals Unprovoked Attacks and Dynamic Assembly
Source: Cell Rep. 2015 Sep 17;12(12):2131–42. doi: 10.1016/j.celrep.2015.08.053 (PMC4594159; doi:10.1016/j.celrep.2015.08.053)
Supplement: Document S2. Article plus Supplemental Information [file mmc5.pdf]

Cell Reports

Article

Visualization of the *Serratia* Type VI Secretion System Reveals Unprovoked Attacks and Dynamic Assembly

Graphical Abstract

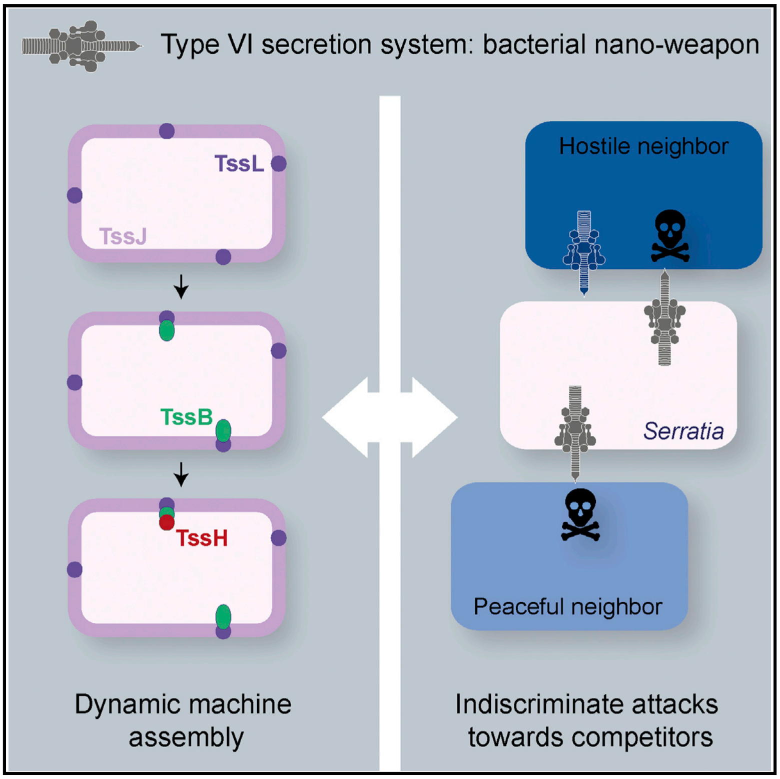

Authors

Amy J. Gerc, Andreas Diepold, Katharina Trunk, ..., Judith P. Armitage, Nicola R. Stanley-Wall, Sarah J. Coulthurst

Correspondence

s.j.coulthurst@dundee.ac.uk

In Brief

Gerc et al. describe the use of fluorescence microscopy to visualize the bacterial type VI secretion system in vivo. They show that *Serratia* can indiscriminately fire this system without a trigger from a neighboring cell and observe distinct and dynamic behaviors of different core components of the system.

Highlights

- T6SSs are used by bacteria to attack competitors
- Fluorescence microscopy has been used to visualize a functioning T6SS in vivo
- *Serratia* T6SS fires without a cell contact trigger, targeting even peaceful neighbors
- Distinct behavior of different T6SS components highlights dynamic machine assembly

Gerc et al., 2015, Cell Reports *12*, 2131–2142
September 29, 2015 ©2015 The Authors
http://dx.doi.org/10.1016/j.celrep.2015.08.053

OPEN ACCESS
CellPress

Visualization of the *Serratia* Type VI Secretion System Reveals Unprovoked Attacks and Dynamic Assembly

Amy J. Gerc,[1,5] Andreas Diepold,[2,5] Katharina Trunk,[1] Michael Porter,[3] Colin Rickman,[4] Judith P. Armitage,[2] Nicola R. Stanley-Wall,[1] and Sarah J. Coulthurst[1,*]
[1]Division of Molecular Microbiology, College of Life Sciences, University of Dundee, Dow Street, Dundee DD1 5EH, UK
[2]Department of Biochemistry, University of Oxford, South Parks Road, Oxford OX1 3QU, UK
[3]Centre of Gene Regulation and Expression, College of Life Sciences, University of Dundee, Dow Street, Dundee DD1 5EH, UK
[4]Edinburgh Super-Resolution Imaging Consortium, www.esric.org, and Institute of Biological Chemistry, Biophysics and Bioengineering, Heriot-Watt University, Edinburgh EH14 4AS, UK
[5]Co-first author
*Correspondence: s.j.coulthurst@dundee.ac.uk
http://dx.doi.org/10.1016/j.celrep.2015.08.053
This is an open access article under the CC BY license (http://creativecommons.org/licenses/by/4.0/).

SUMMARY

The Type VI secretion system (T6SS) is a bacterial nanomachine that fires toxic proteins into target cells. Deployment of the T6SS represents an efficient and widespread means by which bacteria attack competitors or interact with host organisms and may be triggered by contact from an attacking neighbor cell as a defensive strategy. Here, we use the opportunist pathogen *Serratia marcescens* and functional fluorescent fusions of key components of the T6SS to observe different subassemblies of the machinery simultaneously and on multiple timescales in vivo. We report that the localization and dynamic behavior of each of the components examined is distinct, revealing a multi-stage and dynamic assembly process for the T6SS machinery. We also show that the T6SS can assemble and fire without needing a cell contact trigger, defining an aggressive strategy that broadens target range and suggesting that activation of the T6SS is tailored to survival in specific niches.

INTRODUCTION

Bacterial cells utilize diverse and often sophisticated mechanisms to adapt to, and manipulate, their environment, including co-operative and competitor organisms. Protein secretion systems are widely used to interact with abiotic environments, host eukaryotic organisms, and other bacteria. These are specialized machineries for translocating particular proteins to the exterior of the bacterial cell or directly into other cells, and thus represent critical determinants of bacterial pathogenicity and competitive fitness. Multiple classes of secretion system have been identified, with distinct mechanisms of membrane translocation (Kuhn, 2014). One of the most recently described, the Type VI secretion system (T6SS), is becoming increasingly recognized as a widespread and important weapon in the armory of varied Gram-negative bacterial pathogens and symbionts. T6SSs can act as classical virulence factors by injecting toxic effector proteins into eukaryotic cells, including actin modification or phospholipase enzymes (Durand et al., 2014). However, it is now clear that a major function, perhaps the primary function, of T6SSs is to attack competitor bacterial cells and thus promote the fitness of the secreting cell in polymicrobial infection sites and other bacterial communities (Russell et al., 2014). Hence, anti-bacterial T6SSs represent key indirect virulence factors. Anti-bacterial T6SSs can inject multiple distinct anti-bacterial toxins into target cells, causing efficient killing of competitor bacteria. These toxic effectors include peptidoglycan hydrolases, phospholipases, and nucleases, which attack the cell wall, membrane, and nucleic acid, respectively, of target cells. Secreting cells possess specific immunity proteins for each effector. These immunity proteins are able to neutralize the cognate toxin in order to prevent self-toxicity or intoxication by neighboring siblings (Durand et al., 2014; Russell et al., 2014).

The T6SS is a large macromolecular assembly spanning the bacterial cell envelope and whose mode of action is related to the injection mechanism of contractile bacteriophage tails. Recent work has revealed key aspects of the organization and mechanism of the T6SS, but the picture is far from complete. According to current models (Ho et al., 2014; Zoued et al., 2014), the T6SS is built using fourteen "core" components that form several subassemblies. An extracellular puncturing device, which is fired from the cell, is made up of a tube of Hcp (TssD) with a trimer of VgrG (TssI) at its distal end, further sharpened by a PAAR protein at the tip (Brunet et al., 2014; Shneider et al., 2013). A membrane complex, made up of the integral inner membrane proteins TssL and TssM and the outer membrane lipoprotein TssJ, anchors a cytoplasmic baseplate-like structure at the cell envelope. Upon this basal complex, a contractile tubular sheath made of TssBC subunits assembles in the cytoplasm, around the Hcp-VgrG structure (Basler et al., 2012; Brunet et al., 2014; Zoued et al., 2014) (Figure 1A). Prior to firing,

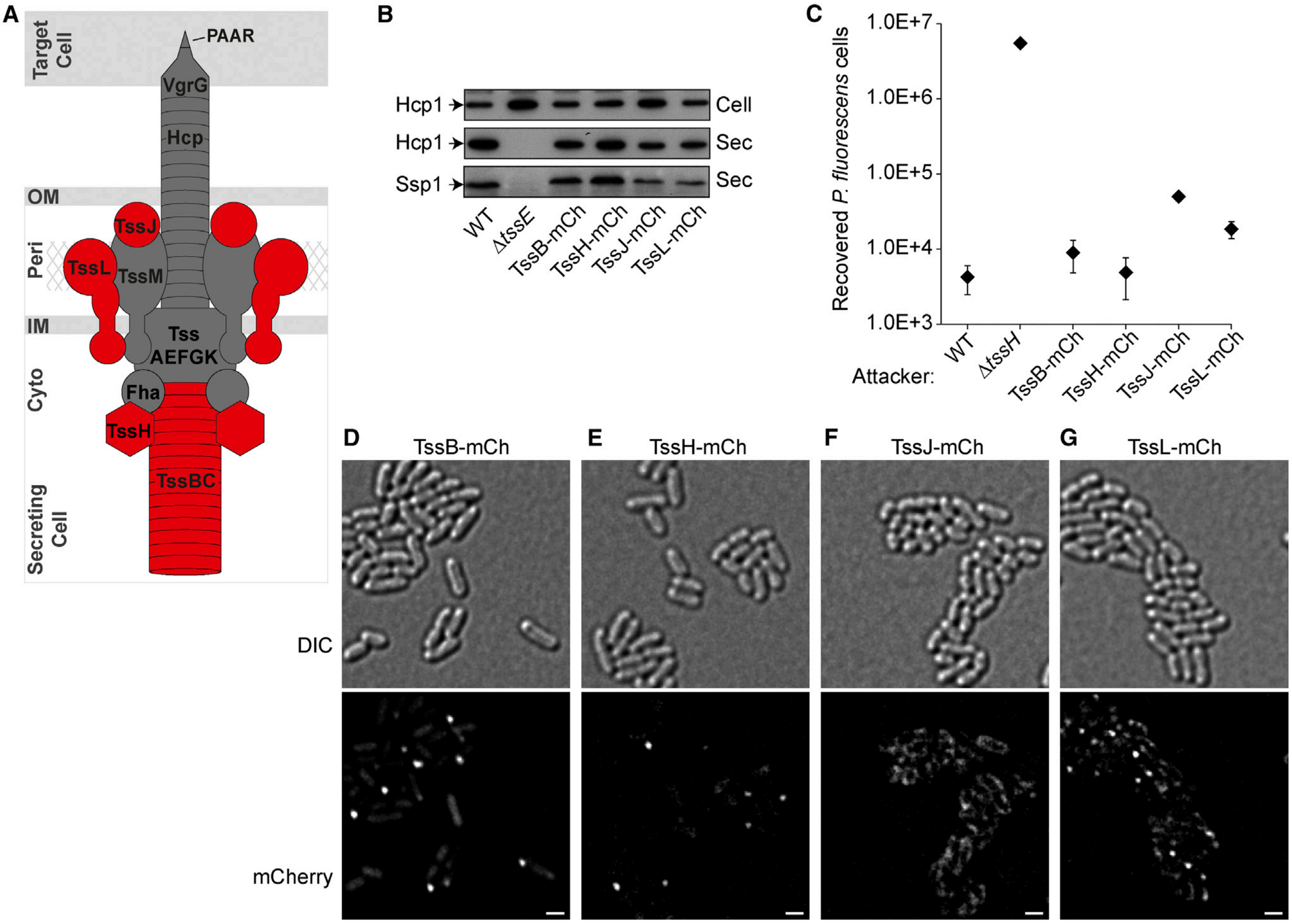

Figure 1. Visualization of Distinct Components within an Active Type VI Secretion System in *Serratia marcescens*

(A) Cartoon depiction of the T6SS with the components visualized in this study highlighted in red. The fourteen core components and one accessory component, Fha, are labeled. Cytoplasm (cyto), periplasm (peri), inner membrane (IM), and outer membrane (OM) of the secreting cell are indicated.

(B) T6SS-dependent secretion of Hcp and the effector Ssp1 by *S. marcescens* Db10 (WT) and derivatives expressing fusions of mCherry to the C terminus of TssB (TssB-mCh), TssH (TssH-mCh), TssJ (TssJ-mCh), or TssL (TssL-mCh). The T6SS inactive mutant Δ*tssE* is a negative control, and cellular (cell) and secreted (sec) fractions were subjected to immunoblotting using anti-Hcp and anti-Ssp1 antisera as indicated.

(C) T6SS-dependent antibacterial activity of fluorescent reporter strains against *P. fluorescens* target cells. Recovery of target cells following a 4-hr co-culture with the attacking strains of *S. marcescens* indicated; points show mean ± SEM (n = 4).

(D–G) Representative images of cells expressing TssB-mCh (D), TssH-mCh (E), TssJ-mCh (F), or TssL-mCh (G). Upper panels: DIC images; lower panels: corresponding fluorescence images (mCherry channel); scale bar, 1 μm.

See also Figure S1.

this TssBC sheath is in an extended conformation. Contraction of the TssBC sheath then propels the puncturing device through the basal complex, out of the cell, and into an adjacent target cell. The contracted TssBC sheath is recognized by the AAA+ ATPase, TssH (ClpV), which disassembles the sheath, allowing recycling of the TssBC subunits and the components of the basal complex (Basler and Mekalanos, 2012; Kapitein et al., 2013; Kube et al., 2014). Effectors are translocated by covalent or non-covalent association with different components of the puncturing device (Dong et al., 2013; Shneider et al., 2013; Silverman et al., 2013; Whitney et al., 2014).

Visualization of TssB-sfGFP (also known as VipA-sfGFP) foci in *Vibrio cholerae* first revealed dynamic cycles of "firing" by the T6SS, with cycles of extension (assembly), rapid contraction, and disassembly of the TssBC sheath being observed (Basler et al., 2012). TssH has also been reported to form dynamic foci, which correspond to its association with the contracted TssB sheath and thus, like TssB foci, report firing of the T6SS (Basler et al., 2013; Basler and Mekalanos, 2012). A striking regulatory strategy has been observed for the H1-T6SS of *Pseudomonas aeruginosa*, termed "Tit-for-Tat" (Basler et al., 2013). Incoming T6SS attacks are sensed by a post-translational regulatory cascade, resulting in the assembly of an active T6SS at the point of attack and a retaliatory strike back toward the attacking cell. As a result, adjacent *P. aeruginosa* cells can be observed to "duel" with each other, with a TssH-GFP focus from each cell "paired" at the interface between the neighbors, whereas almost no activity is observed against bacterial cells lacking a T6SS.

OPEN ACCESS
CellPress

However, it is currently unknown whether this defensive strategy, or contact-dependent activation in general, is typical among related T6SSs in other organisms.

To provide a broader perspective on T6SS activation and insight into T6SS assembly and function, we considered the T6SS in the opportunistic pathogen *Serratia marcescens*. *S. marcescens* is a highly versatile organism found in many environmental niches but particularly known for its ability to be a potent insect pathogen and to cause hospital-acquired infections (Iguchi et al., 2014; Mahlen, 2011). Indeed, it is a typical representative of the clinically significant class of antibiotic-resistant opportunistic Enterobacteriaceae. The disparate and opportunistic lifestyles of *S. marcescens* suggest a need for efficient competitive strategies against other bacteria, and it is known to produce several antimicrobial agents, in some cases responding to other bacterial cells at a distance (Petersen and Tisa, 2013). We have shown that *S. marcescens* Db10 possesses a single T6SS with potent anti-bacterial activity, delivering at least six anti-bacterial effector proteins, including the peptidoglycan hydrolases Ssp1 and Ssp2 (English et al., 2012; Fritsch et al., 2013; Murdoch et al., 2011; Srikannathasan et al., 2013).

In this study, we examined the dynamic behavior and activation of the *S. marcescens* Db10 T6SS at the single-cell level. Using fluorescence microscopy, we observed the distribution, mobility, and localization of core components of the machinery. In particular, we analyzed TssL and TssJ, since the membrane complex had never before been visualized in vivo and the behavior of its constituents relative to other T6SS components was unknown. Our results reveal that the T6SS in *S. marcescens* does not show defensive "Tit-for-Tat" behavior but instead acts aggressively, exhibiting random, non-contact-dependent firing. Further, we show that four core T6SS components, TssB, TssH, TssJ, and TssL, all exhibit distinct behavior in vivo and provide support for a model of T6SS assembly, whereby the contractile sheath assembles at a subset of potential sites defined by the membrane complex in anticipation of firing.

RESULTS

Visualization of Four Core Components of the *S. marcescens* T6SS within the Context of a Functional Machinery Reveals Distinct Patterns of Localization

To study the T6SS in a physiologically relevant manner, we constructed several reporter strains of *S. marcescens* Db10 with mCherry fused to the C terminus of the T6SS component of interest, encoded at the native chromosomal location. Using this approach, the fusion protein should be expressed at the normal level in concert with the other components of the machinery. The first T6SS components chosen were the sheath protein TssB and the sheath depolymerase TssH (ClpV). These cytoplasmic components have been studied previously by microscopy and thus provide a reference point and allow comparison with different T6SSs. In contrast, the membrane subcomplex of the T6SS has never been studied microscopically and its behavior relative to the sheath components is entirely unknown. Therefore, the inner membrane protein TssL and the outer membrane lipoprotein TssJ were selected for study. The predicted location of each of these components within the T6SS is illustrated in Figure 1A. Following construction of the TssB-mCh, TssH-mCh, TssL-mCh, and TssJ-mCh strains, the functionality of their T6SSs was assessed. All four strains were able to secrete Hcp and the effector protein Ssp1 (Figure 1B), confirming that the basic function of the system had been preserved. A more sensitive assay for full T6SS function is quantitative determination of T6SS-dependent anti-bacterial activity. Against *P. fluorescens* target cells, the TssB-mCh and TssH-mCh strains showed wild-type (WT) killing activity (Figure 1C). The TssL-mCh and TssJ-mCh strains showed a modest decrease in killing efficiency compared with the wild-type but still showed considerable anti-bacterial activity (a several 100-fold reduction in target cell recovery compared with a T6SS mutant attacker). Immunoblotting further confirmed that full-length fusion proteins were being produced (Figure S1).

Examination of each reporter strain using fluorescence microscopy revealed distinct distributions. As expected, TssB-mCh formed bright foci in a proportion of the cells, with diffuse cytoplasmic fluorescence also visible in most cells (Figure 1D). Similarly, TssH-mCh formed readily visible foci in a subpopulation of the cells (Figure 1E). In contrast, TssJ-mCh did not form foci but was unevenly distributed around the periphery of the cells, in the region of the cell envelope (Figure 1F). The distribution of TssL-mCh was different again, with a mixture of foci and "patchy" fluorescence tending to the periphery of the cells (Figure 1G).

The *S. marcescens* T6SS Is Active throughout Growth of a Microcolony and Does Not Depend on Cell-Cell Contact for Activation

Focus formation by TssB-fluorophore fusion proteins has been used to monitor active or "firing" T6SSs in several organisms (Basler et al., 2012; Brunet et al., 2013). While the TssB-mCh foci we observed in *S. marcescens* showed some variation in size and shape, we were unable to clearly divide them into the two classes "extended" (primed to fire) and "contracted" (just fired) as has been reported for TssB-sfGFP in *V. cholerae* (Basler et al., 2012). Nevertheless, it was readily apparent that TssB focus formation was dynamic on a timescale of minutes, with foci appearing and disappearing in different cells throughout a population. Time-lapse imaging over 6 hr allowed observation of TssB foci throughout the development of a microcolony from a single founder cell (Figure 2; Movie S1). The subpopulation of cells exhibiting TssB-mCh foci changes every 10 min (Figure 2B), indicating that cycles of sheath extension, contraction, and disassembly occur within minutes.

The frequency of T6SS assembly and firing, reported by formation of TssB or TssH foci, and the trigger for activation has been suggested to differ between organisms (Basler et al., 2013). In order to examine these properties for our system, we introduced the TssB-mCh reporter into a strain of *S. marcescens* Db10 uniformly expressing cytoplasmic GFP (TssB-mCh, Δ*lacZ*::GFP). Nearly 4,000 cells were imaged, with a representative partial field of view shown (Figure 3A). The number of cells in each field of view was determined from automated masks applied using the GFP signal, and TssB-mCh foci were manually identified and counted. Considering all cells, the average number of TssB foci per cell, at a given instant, was 0.35. 69% of cells

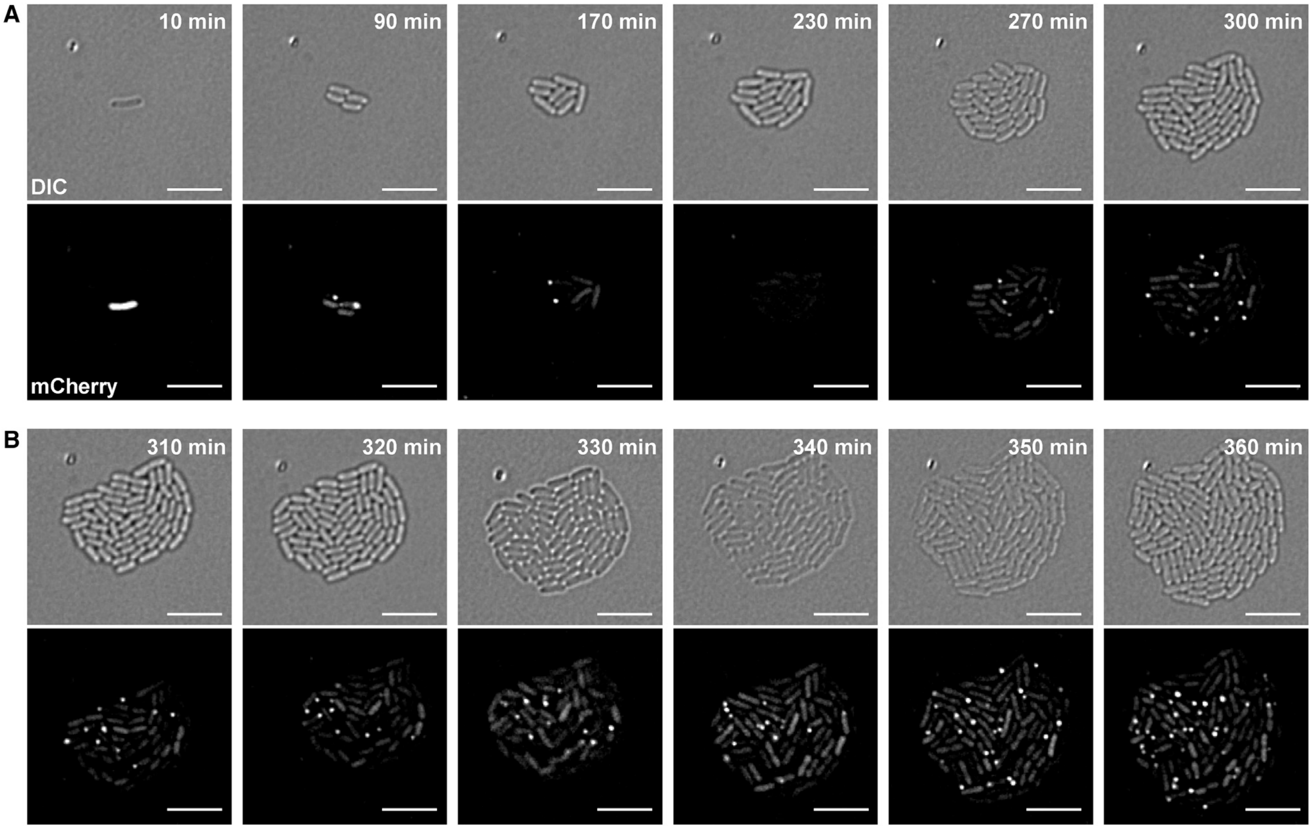

Figure 2. TssB Foci Exhibit Dynamic Behavior throughout the Growth of a Microcolony

(A and B) Time-lapse imaging of TssB-mCh every 10 min over a 6-hr period. DIC (upper) and fluorescence (lower) images are shown for selected time points during the first 5 hr (A) and every 10 min over the final hour (B). Scale bar, 5 μm. The full series is shown in Movie S1.

had no foci, 28% had one, 3% had two, and just 0.2% (seven cells) had three (Figure 3B). Specifically considering isolated single cells with no touching neighbors, the number of foci per cell was 0.53, with 54% of cells having no foci, 39% having one focus, and 7% having two. Thus, it is clear that focus formation, and by implication T6SS activation, in *S. marcescens* is not dependent on cell-cell contact. In addition, a lack of any observable tendency for foci to be "paired" in neighboring cells (i.e., no "dueling") implies at the single-cell level that there is no "Tit-for-Tat" strategy operating in *S. marcescens*. To further confirm that *S. marcescens* does not utilize this defensive regulatory strategy, we demonstrated that *S. marcescens* Db10 shows indistinguishable T6SS-dependent killing of T6SS-inactive versus T6SS^{+} *S. marcescens* ATCC274 target cells (Figure 3C). Hence, *S. marcescens* is aggressive toward even non-attacking target cells, in stark contrast with *P. aeruginosa*, which utilizes the defensive "Tit-for-Tat" strategy and therefore does not efficiently kill T6SS-deficient bacteria (Basler et al., 2013).

Distribution and Mobility of Different T6SS Components within the Cell

Having established the overall behavior of our T6SS at the single-cell level using the relatively well-characterized TssB protein, we compared the properties of the other fusion proteins with TssB-mCh. As above, initial observation using "snapshot" imaging revealed distinct localization patterns for each protein (Figures 1D–1G). To examine the localization of the different T6SS components over different timescales, we used fluorescence microscopy to visualize bacteria expressing the functional mCherry-labeled components with frame rates of 100 ms, 1 s, and 10 s. TssB foci (Figure 4A) were stable and immobile on short timescales (up to a few seconds), while some changes in both the intensity and the localization could be observed over 10-s intervals (bottom row). TssL was found to be more dynamic than TssB (Figure 4B). Interestingly, while the positions of TssL spots remained relatively fixed, especially for brighter foci, spot intensities fluctuated over the 100-ms range, especially in weaker spots. TssH is even more dynamic than TssB and TssL (Figure 4C). However, while there is considerable movement for diffuse TssH and small foci, bright foci could be stable for more than 10 s (bottom row). Strikingly, the relatively weak TssJ patches moved considerably over seconds (Figure 4D). Of note, for TssB and TssH, cases were observed in which bright foci appeared and disappeared in multiple cycles. While TssB foci tended to disappear after 1 to 2 min and form at another position (Figure 4E; Movie S2), bright TssH foci were sometimes found to cycle between focal and diffuse fluorescence with a period of about 50 s for each state (Figure 4F; Movie S3).

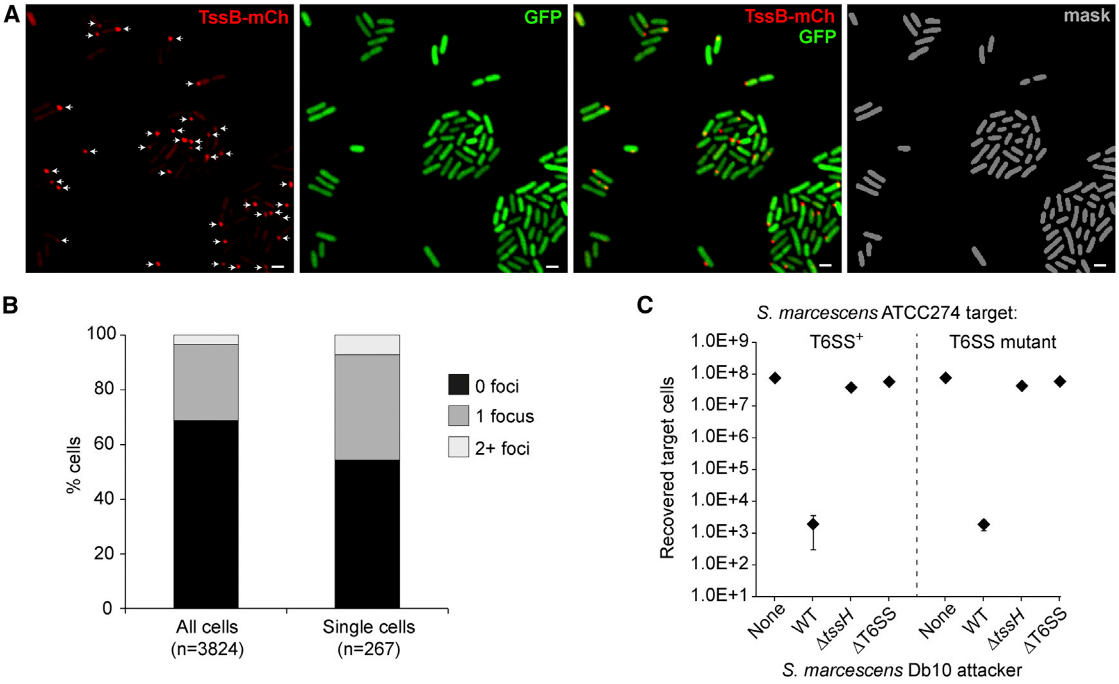

Figure 3. The *S. marcescens* T6SS Does Not Require Cell-Cell Contact for Activation and Does Not Exhibit "Dueling" Behavior

(A and B) Analysis of *S. marcescens* Db10 expressing the TssB-mCh reporter fusion together with uniform cytoplasmic GFP. (A) Part of a representative field of view showing the red fluorescence channel (TssB-mCherry), green fluorescence channel (constitutive cytoplasmic GFP), merged red/green, and the automatically generated GFP mask. Manually identified TssB-mCh foci are highlighted with white arrows; scale bar, 1 μm. (B) Percentage of cells with 0, 1, or 2+ foci, either within the whole population (all cells) or within the subgroup of isolated cells with no touching neighbors (single cells).

(C) T6SS-mediated killing of wild-type ($T6SS^{+}$) or Δ*tssE* (T6SS mutant) *S. marcescens* ATCC274 target cells by different attacker strains of *S. marcescens* Db10, as indicated. WT, wild-type; Δ*tssH*, T6SS inactive mutant; ΔT6SS, mutant lacking entire T6SS; none, media only; points show mean ± SEM (n = 4).

Localization of each of the fusion proteins was also determined in a Δ*tssE* mutant background, where absence of the essential baseplate component TssE results in an inactive T6SS. TssB and TssH no longer formed foci in the Δ*tssE* mutant, consistent with a lack of sheath assembly and contraction. In contrast, the localization of TssJ-mCh and TssL-mCh was unchanged (Figure S2). Formation of foci by TssL in a similar manner in both wild-type and Δ*tssE* backgrounds implies that TssL localization is not dependent on assembly of the baseplate.

Co-localization Analysis Reveals Non-reciprocal Associations between Different T6SS Core Components

To directly compare the localization of TssH, TssJ, and TssL with the reference TssB, functional dual reporter strains were constructed. The chromosomally encoded TssH-mCh, TssJ-mCh, and TssL-mCh fusions were each combined with a *tssB-GFP* allele for co-expression of a TssB-GFP fusion protein from the normal chromosomal location. As for the single TssB-,TssH-, TssJ-, and TssL-mCh fusion strains, strains of *S. marcescens* Db10 expressing TssB-GFP alone or any of the TssH-,TssJ-, TssL-mCh, or TssB-GFP dual reporters retained the ability to secrete Hcp, confirming their T6SS functionality (Figure 5A). Imaging the three dual reporter strains revealed distinct patterns of association with TssB foci (Figure 5B). TssH foci were found to be strongly co-localized with TssB foci, whereas TssB foci were often found without corresponding TssH foci (top row). TssJ is localized much less specifically around the cell, and specific co-localization of TssJ with TssB was not observed (middle row), although TssJ sometimes appeared enriched around TssB foci. The association between TssL and TssB followed a different pattern again. TssB foci were highly co-localized with TssL foci, but many additional TssL foci without TssB were also observed (bottom row). Quantitative co-localization by a Pearson's correlation method (comparing each channel over every pixel) gave similar correlation values (between 0.48 and 0.58) for each of TssH, TssJ, and TssL with TssB, presumably because all show some diffuse non-focal fluorescence in addition to any foci. However, given that the foci are of primary interest, a more suitable, object-based approach was adopted.

Object-based co-localization was performed between TssB-GFP and either TssH-mCh or TssL-mCh, based on measuring the distance between the center of each red focus and center of the nearest green focus, or vice versa. If the distance is <0.272 μm, any difference in the locations of the two foci is below the resolution limit of the microscope and the original foci show significant overlap ("co-localized"). Focus detection and co-localization analysis was automated using custom algorithms (see the Experimental Procedures), and an example of the foci detected for a representative field of view is shown in Figure 6A. TssJ-mCh was not included in this analysis since clear foci could not be detected. Figure 6B shows the frequency distribution of the distances between TssH/L-mCh foci and their

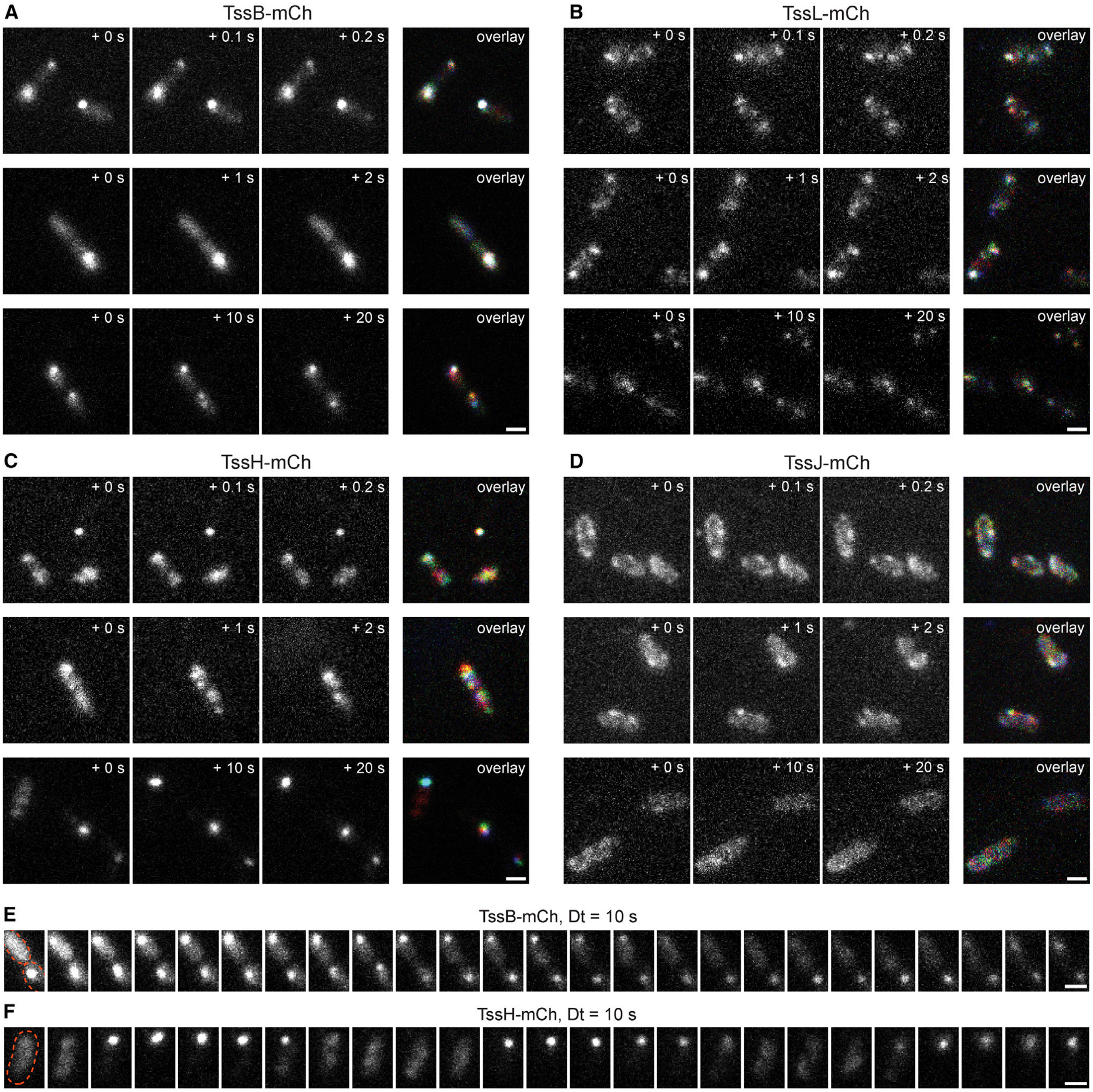

Figure 4. TssH, TssJ, and TssL Show Differential Distribution and Mobility from TssB and Each Other

(A–D) Representative set of fluorescence images of TssB-mCh (A), TssL-mCh (B), TssH-mCh (C), and TssJ-mCh (D) acquired at 100-ms (top), 1-s (middle), or 10-s intervals (bottom). From left to right: three sequential frames and an overlay are shown. In the overlay, the signal from the three frames is colored consecutively red, green, and blue; any signal present in all three frames will appear white, whereas movement will result in the appearance of color.

(E and F) Example time courses of TssB-mCh (E) and TssH-mCh (F) acquired over a longer time; the cells shown in lower panels of parts A and C, respectively, are shown in 10-s intervals over a total of 4 min. Images were manually corrected for xy drift and photobleaching with the outline of the cell shown in red in the first image. The corresponding uncropped series of raw images are shown as Movies S2 and S3. Scale bars, 1 μm.

See also Figure S2.

nearest TssB-GFP foci and between TssB-GFP foci and their nearest TssH/L-mCh foci. These data are summarized in Figures 6C and 6D and show good agreement with the qualitative observations. Comparing TssH with TssB, in total there were 2-fold fewer TssH foci per cell than TssB. The TssH foci showed a high frequency of co-localization with TssB foci, whereas TssB showed a lower frequency of co-localization with TssH, resulting in nearly two-thirds of TssB foci not having TssH foci associated

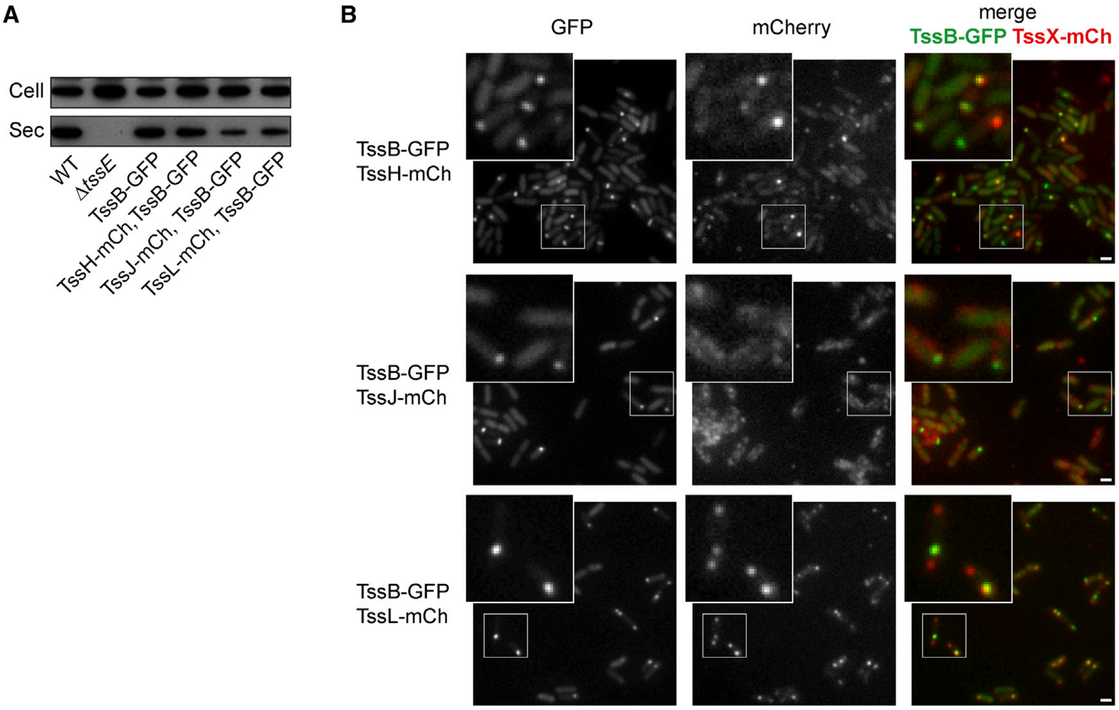

Figure 5. Simultaneous Observation of Two Type VI Components Reveals Differing Associations of TssH, TssJ, and TssL with TssB

(A) Hcp secretion by *S. marcescens* Db10 (WT) and derivatives expressing a fusion of GFP to the C terminus of TssB (TssB-GFP), or expressing both TssB-GFP and a fusion of mCherry with TssH (TssH-mCh, TssB-GFP), TssJ (TssJ-mCh, TssB-GFP), or TssL (TssL-mCh, TssB-GFP). Cellular (cell) and secreted (sec) fractions subjected to anti-Hcp immunoblot, with the Δ*tssE* mutant as a negative control.

(B) Representative fluorescence images from cells expressing TssH-mCh and TssB-GFP (top), TssJ-mCh and TssB-GFP (middle), or TssL-mCh and TssB-GFP (bottom). Indicated parts of the micrographs are magnified in the insets. Panels from left to right: GFP (TssB-GFP) channel, mCherry (TssX-mCh) channel, and a false-colored merge. Scale bars, 1 μm.

(Figures 6B–6D). In contrast, there were almost 3-fold more TssL foci than TssB foci, and this time there was a high frequency of co-localization of TssB with TssL but a much lower co-localization of TssL with TssB. In other words, opposite to the situation with TssH, the majority of TssB foci are associated with TssL foci, whereas only a minority of TssL are associated with TssB. Overall, these data demonstrate that a subpopulation of TssL foci are occupied by TssB, and in turn, a subpopulation of all TssB foci contain TssH. The data imply that simultaneous co-localization of all three proteins can occur, but it has not been directly visualized here.

DISCUSSION

In this work we observed core proteins of the *S. marcescens* T6SS in vivo using fluorescence microscopy. Importantly, these observations are physiologically relevant since all fusion proteins were functional, expressed from the native chromosomal location and in a wild-type genetic background. By examining T6SS behavior on multiple timescales, from milliseconds to hours, we observed different dynamics between scales and components and discovered that each of the four key components behaves in a distinct manner, providing important insight into the assembly and function of the T6SS. Additionally, we provide support for the idea that the trigger for activation of a particular T6SS differs between even related T6SSs, emphasizing the versatility of this system.

Time-lapse imaging of TssB showed that the *S. marcescens* T6SS is active from a single cell through rounds of division to a microcolony containing hundreds of cells. Work in *V. cholerae* has indicated that extension of the TssB sheath to form a focus takes around 30 s and then the sheath may remain in this state for up to some minutes until a rapid contraction event (<5 ms) occurs, followed by disassembly over the next 30–60 s (Basler et al., 2012). These parameters are consistent with our observation that the subpopulation of cells displaying a focus has changed every 10 min in *S. marcescens* (Figure 2B) and with observations of cycles of TssB and TssH focus formation with a period of 1 to 2 min (Figures 4E and 4F). Given that approximately one-third (31%) of *S. marcescens* cells at any given instant display at least one TssB focus and thus have an active T6SS (immediately pre- or post-firing), this suggests that each cell in the population will fire multiple times over a few hours, helping to explain how competitors in a mixed population can be almost eliminated on such timescales (e.g., Figure 3C). By quantifying the overall frequency of TssB foci per cell as 0.35 and using dual reporter strains to determine the number of TssH and TssL foci per cell relative to TssB, we estimate that there are around 0.2 TssH foci per cell and 0.9 TssL foci per cell in *S. marcescens*. The number of TssH foci per cell for

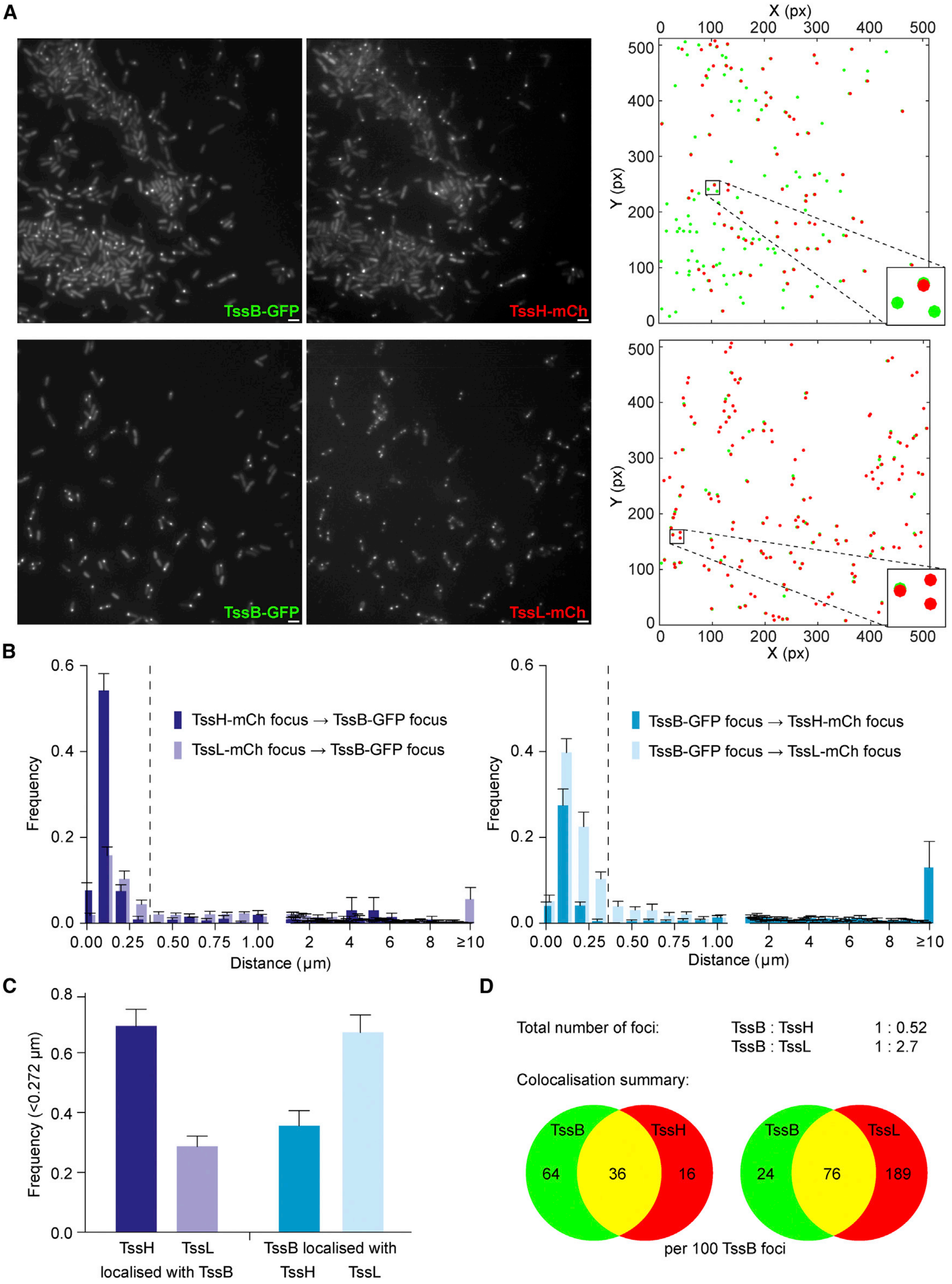

Figure 6. Quantitative Co-localization Analysis Demonstrates Non-reciprocal Preferences for TssH Foci to Co-localize with TssB Foci and for TssB Foci to Co-localize with TssL Foci

(A) Fluorescence microscopy images (TssB-GFP, left; TssX-mCh, middle) and map of automatically detected foci (right) of a representative field of view for *S. marcescens* co-expressing TssH-mCh and TssB-GFP (upper) or TssL-mCh and TssB-GFP (lower). On the map, GFP foci are shown in green and mCherry in red over the top. A small region of each map is magnified in the inset. Scale bars, 2 μm.

(legend continued on next page)

P. aeruginosa H1-T6SS was reported to be similar (0.15) (LeRoux et al., 2012), whereas the number of TssB and TssH foci in *V. cholerae* can be significantly higher (Basler and Mekalanos, 2012; Basler et al., 2012; Borgeaud et al., 2015). Hence, the number of active T6SSs per cell is not necessarily conserved across different systems. Interestingly, ClpV-5 (TssH) from an anti-eukaryotic T6SS in *Burkholderia thailandensis* exhibits quite different behavior from other T6SSs examined to date. Formation of foci by ClpV-5 is induced following contact with macrophages and the foci are much less dynamic and show pronounced polar localization, perhaps reflecting adaptation of the T6SS to promote establishment of prolonged contact with a eukaryotic cell (Schwarz et al., 2014).

Significantly, we determined that the frequency of T6SS focus formation in *S. marcescens* Db10 is not reduced, and indeed may be increased, in isolated single cells. Hence, the signal triggering T6SS assembly is not cell contact. This observation and the lack of any tendency for foci to be "paired" in adjacent cells demonstrates at the single-cell level that there is no "Tit-for-Tat" regulation in this organism. In *P. aeruginosa*, membrane breach by an incoming T6SS is sensed by the TagQRST complex, which signals to the protein threonine kinase PpkA, resulting in phosphorylation of Fha and assembly of an actively firing T6SS (Basler et al., 2013; Casabona et al., 2013; Mougous et al., 2007). The *S. marcescens* T6SS is related to H1-T6SS of *P. aeruginosa* and also has homologs of Fha, PpkA, and the antagonistic phosphatase PppA. Earlier work in vitro, based on analyzing secretion and protein phosphorylation at the population level, showed that phosphorylation of Fha by PpkA is essential for T6SS activity in *S. marcescens* but suggested that the signal for T6SS activation might not be cell-contact dependent (Fritsch et al., 2013). Further proof for the lack of any "Tit-for-Tat" regulation is provided in the current work by showing that *S. marcescens* exhibits no deficit in killing ability against target bacteria with no T6SS counterattack (Figure 3C). Critically, adoption of an "aggressor" strategy considerably broadens the target range of the *S. marcescens* T6SS. It allows this organism not only to kill prey bacteria with an antibacterial T6SS or other incursive strategy but also to efficiently kill those without a T6SS (as we have previously observed; Murdoch et al., 2011). Of course, the aggressor strategy also provides a temporal advantage: by not waiting for incoming attacks, *S. marcescens* can impede competitors before being damaged itself. We speculate that use of this strategy reflects the fact that normal niches for *S. marcescens* are highly competitive polymicrobial environments, for example, soil. In this case, PpkA may provide the ability to respond to an "off" signal, under specific circumstances in which an active T6SS would be unfavorable. It has been reported that *V. cholerae* does not "duel" either, consistent with its lack of the PpkA/PppA phosphorylation system (Basler and Mekalanos, 2012); "Tit-for-Tat" may in fact be the exception rather than the rule for the T6SS.

Examination of selected individual components of the basic secretion machinery allowed us to observe TssL and TssJ at the single-cell level and also to compare the in vivo behavior of these membrane complex components with that of sheath-associated components, namely, TssB and TssH. This revealed that the behavior of each component is distinct, suggesting a multi-stage, and probably dynamic, assembly process for the T6SS machinery. As expected, TssH formed bright foci similar to TssB and the majority of TssH foci were associated with a TssB focus. This is in agreement with the previous observation of co-localization of TssH with TssB in contracted sheaths in *V. cholerae* (Basler and Mekalanos, 2012). Here, we further provided quantitative data showing that, at any moment, approximately one-third of TssB foci are associated with TssH. These are predicted to be post-firing contracted sheaths in the process of disassembly. On the other hand, around two-thirds of TssB foci are not associated with TssH and thus should be assembled sheaths primed to fire. So, on average, the T6SS spends around twice as long in the extended/primed state than it does contracted. A greater degree of spatial mobility was observed for TssH compared with the TssB foci which, once formed, are stable and immobile over seconds. It would be expected that the complete T6SS machinery, containing an assembled TssBC sheath, should be relatively static and stable, both due to its large size and the need to physically propel the puncturing device out from the cell. The considerable movement of diffuse and small foci of TssH may represent dynamic behavior in finding and "loading on" to the contracted sheath, but could also reflect small accumulations of TssH in the cytoplasm, either completing depolymerization of contracted sheaths or removing non-productive sheaths forming aberrantly without a basal complex (Kapitein et al., 2013). Indeed, a small population of TssH foci not associated with TssB was suggested by our analysis. It has been suggested that in a subset of T6SSs, including that of *S. marcescens* and the *P. aeruginosa* H1-T6SS, sheath disassembly may be more complex than in *V. cholerae*, with the involvement of the accessory protein TagJ in recruitment of TssH to contracted TssBC (Förster et al., 2014). The authors proposed that TagJ might be particularly important in mediating depolymerization of small TssBC fragments in the cytoplasm after initial disruption of the intact sheath. Thus, TssH foci not associated with bright TssB foci might instead represent TagJ-TssH complexes completing the final breakdown of spent sheaths.

This study revealed that the inner membrane protein TssL also forms readily detectable foci, together with less-defined areas of fluorescence. For the large majority of TssB foci, a co-localized TssL focus is also detected, suggesting that TssB sheaths assemble on a basal complex containing multiple copies of

(B) Frequency histograms of the distances between TssH-mCh or TssL-mCh foci and their nearest TssB-GFP foci (left), or between TssB-GFP foci and their nearest TssH-mCh or TssL-mCh (right); cumulative data from 11 (TssH) or 10 (TssL) fields of view; bars show mean ± SEM. A dashed line indicates the 0.272-μm colocalization threshold.

(C) Overall frequencies of co-localization (foci < 0.272 μm apart) of TssH and TssL foci with TssB foci, or vice versa.

(D) Venn diagram summarizing the observed co-localization between TssB and TssH (left), or between TssB and TssL (right); the total number of TssH and TssL foci relative to number of TssB foci is given as a ratio.

TssL. Indeed, TssM, the other inner membrane protein with which TssL tightly associates (Ma et al., 2009), is required for TssB sheath assembly (Kapitein et al., 2013). However, many more TssL than TssB foci are observed, with most not having a co-incident TssB focus, and TssL foci can be noticeably more mobile than TssB. These observations suggest that pre-existing TssL-containing complexes may sit in the membrane ready to form sites for assembly of the contractile part of the machinery, a suggestion supported by the observation that formation of TssL foci is independent of the baseplate component TssE. This could parallel the case in, for example, Type II secretion systems, where localization of the outer membrane secretin is believed to define the site of assembly (Lybarger et al., 2009). In contrast with the other components, the outer membrane lipoprotein TssJ is dispersed around the outside of the cell without forming foci and is very mobile. This reveals behavior distinct from that of the inner membrane components TssLM, although at some point during the T6SS assembly and firing cycle TssJ should join the membrane complex since it interacts with TssM (Felisberto-Rodrigues et al., 2011). This interaction may be transient or only involve a small fraction of TssJ proteins, since no redistribution to form visible foci was detected. We speculate that TssJ might aid non-disruptive passage of the Hcp-VgrG spike through the outer membrane.

In conclusion, our data allow us to propose a model for T6SS assembly whereby TssLM form dynamic potential sites for T6SS assembly at the inner membrane and TssJ is present throughout the outer membrane ready to join the membrane complex during assembly or the firing cycle. Either stochastically or in response to an activating signal, TssB forms a focus at one of these TssLM sites as the sheath polymerizes upon a cytoplasmic baseplate structure and subsequently contracts. Following contraction, TssH associates with the sheath to orchestrate its disassembly, forming visible foci, which may persist for a short time as the final pieces of spent sheath are removed. The T6SS is an impressively effective weapon against competitor bacteria, with a rapid rate of discharge of its deadly cargo into adjacent cells. Indeed, *S. marcescens*, in contrast with *P. aeruginosa*, chooses to fire without waiting for a signal from a potential victim. Such unprovoked attack will broaden its target range and may enhance its fitness in highly competitive niches. It seems clear that both the T6SS itself and its ability to be flexibly deployed will play key roles in determining the composition of many polymicrobial populations, with associated implications for both health and disease.

EXPERIMENTAL PROCEDURES

Bacterial Strains and Strain Construction

Bacterial strains used in this study were wild-type *S. marcescens* Db10 or derivatives thereof and are detailed in Table S1. Routinely, strains of *S. marcescens* were cultured at 30°C with good aeration and in LB media. Strains of Db10 expressing chromosomally encoded mCherry and GFP fusion proteins or uniform cytoplasmic GFP were constructed using allelic exchange based on the suicide vector pKNG101 as described previously (Kaniga et al., 1991; Murdoch et al., 2011). For TssB-, TssJ-, TssH-, and TssL-mCh and TssB-GFP reporter strains, the wild-type alleles were replaced with alleles encoding fusions of mCherry or GFP to the C terminus of the Tss protein, separated by a GAGAPVAT linker, at the normal chromosomal location. In the case of TssB and TssL, to preserve expression of the downstream gene, it was necessary to further fuse a second copy of the final nine amino acids of the original protein to the C terminus of mCherry or GFP. The mCherry gene was amplified from pmCherry-N1 (Clontech) and GFP was the GFPmut2 variant from pBL165 (Stanley et al., 2003). For controllable expression of cytoplasmic GFP, a cassette containing *gfpmut2* under the control of the T5 promoter, together with a Kan^R gene, was used to replace the *lacZ* gene of *S. marcescens* Db10. An in-frame deletion of *tssE* was constructed in *S. marcescens* ATCC274 as described previously (Murdoch et al., 2011). Sm-resistant derivatives of this mutant and wild-type ATCC274 were simultaneously isolated by phage-mediated transduction of the Sm-resistance allele from SJC17 (Murdoch et al., 2011), using Φ3M (Coulthurst et al., 2006). The genome sequence for *S. marcescens* Db11 is used for *S. marcescens* Db10 since they differ by only a point mutation in *rpsL* in Db11 (Iguchi et al., 2014).

Assays for T6SS Function

Anti-Hcp immunoblotting of total cellular and culture supernatant (secreted) protein samples and anti-Ssp1 immunoblotting of secreted proteins was performed after 5 hr growth in LB as described previously (English et al., 2012; Murdoch et al., 2011). Total cellular fractions of strains expressing Tss-mCh/GFP fusion proteins were also probed using mCherry (custom) or GFP (Roche) antibodies to confirm the integrity of the fusion protein. T6SS-mediated antibacterial activity was measured using co-culture (“competition”) assays as described previously (Murdoch et al., 2011). Briefly, attacker strains of *S. marcescens* Db10 were mixed with target strains *P. fluorescens* KT02 or *S. marcescens* ATCC274 strains KT81 or KT82 at an initial ratio of 5:1 and co-cultured for 4 hr at 30°C, followed by enumeration of viable, surviving target cells by selection on streptomycin-containing media.

Fluorescence Microscopy

For microscopy analysis, stationary phase overnight cultures were diluted to an optical density at 600 nm (OD_{600}) of 0.15 in 15–25 ml minimal glucose medium (7 g/l K_2HPO_4, 2 g/l KH_2PO_4 [pH 7], 0.1% [w/v] $(NH_4)_2SO_4$, 0.4 mM $MgSO_4$, 0.2% [w/v] glucose) and incubated for 3.5–4 hr at 30°C with shaking. 1.5 μl of bacterial culture was placed on a microscope slide layered with a pad of minimal glucose medium solidified by the addition of 1.5% agarose. In the case of 6 hr time-lapse imaging (Figure 2), the culture was diluted to an OD_{600} of 0.007 prior to placement on the agarose pad and allowed to equilibrate on the slide for 1 hr prior to imaging. When required, 50 μM IPTG was included to induce expression of cytoplasmic GFPMut2.

For initial analyses of the single Tss-mCh strains and TssB-mCh with cytoplasmic GFP (Figures 1, 2, and 3), images were acquired using a DeltaVision Core wide-field microscope (Applied Precision) mounted on an Olympus IX71 inverted stand with an Olympus 100× 1.4 na lens and Cascade2_512 EMCCD camera (Photometrics), with differential interference contrast (DIC) and fluorescence optics. Datasets (512 × 512 pixels with 13 Z sections spaced by 0.2 μm) were acquired. Images were acquired with an LED-transmitted light source (Lumencor solid state system). GFP and mCherry were detected using a FITC (fluorescein isothiocyanate) filter set (Ex 490/20 nm, Em 528/38 nm) and a TRITC (tetramethylrhodamine) filter set (Ex 555/28, Em 617/73), respectively, with exposure times of 1 s. DIC images were acquired at 32% intensity and exposure times between 25 and 100 ms. Post-acquisition, images were deconvolved using softWoRx and processed using OMERO software (http://openmicroscopy.org) (Allan et al., 2012).

Short time-course microscopy to follow the behavior of Tss-mCh fusion proteins (Figure 4) was performed on a custom-built multi-color fluorescence microscope (Leake et al., 2006; Plank et al., 2009). Fluorescence was excited using a 561-nm laser and fluorescence emission was imaged for an exposure time of 80 ms using a 128 × 128-pixel, cooled, and back-thinned electron-multiplying charge-coupled device camera (iXon DV860-BI; Andor Technology). To reduce photobleaching, the laser was turned off automatically between imaging frames at frame rates of less than 10 Hz. Images were processed and manually adjusted for piezo drift in the xy stage using ImageJ (NIH).

For co-localization analysis (Figures 5 and 6), imaging was performed using a Deltavision Spectris optical sectioning microscope (Applied Precision) equipped with a UPlanSApo 100 ×/1.40 oil objective (Olympus) combined

with 1.6× auxiliary magnification and an Evolve EMCCD camera (Photometrics) was used to take differential interference contrast (DIC) and fluorescence photomicrographs. For fluorophore visualization, either the GFP/hsGFP filter set (Ex 475/28 nm, Em 522/44 nm) or the mCherry/hsCherry filter set (Ex 575/25 nm, Em 634/63 nm) were used. Exposure times were 10 ms for DIC, 1 s for GFP, and 1.5 s for mCherry. Per image, a z stack containing seven frames per wavelength with a spacing of 0.15 μm was acquired. Undeconvolved images were processed with ImageJ (NIH).

Quantitative Image Analysis

For determination of the number of TssB-mCh foci per cell (Figure 3), a total of 50 fields of view from three independent experiments were analyzed. TssB-mCh foci in the TRITC channel were manually identified and counted. The total number of cells in each field of view was determined by automated counting based on detection of uniform cytoplasmic GFP in the FITC channel as follows. Fluorescence images stored in an OMERO database were analyzed in Matlab with the following workflow: each m-by-n z stack (FITC channel) was downloaded into Matlab using the OMERO.matlab toolbox, and the resultant 3D matrix was reshaped into an m-by-n*z plane to minimize edge effects. A normalized threshold for edge detection was calculated by dividing the mean values below the Otsu threshold by the maximum intensity value of the image. The plane was passed through a median filter and an image transform (squaring) emphasized the gradients before edge detection ("Canny" method) was performed using the threshold calculated previously. A diamond structuring element with a radius of one pixel was used to dilate the detected edges before filling enclosed pixels and eroding with the same structuring element. Individual isolated pixels remaining in the image were removed with the "majority" morphological operation. To define cells, the image was reshaped back into an m-by-n z stack (segmentation mask), where each group of connected pixels were given a unique value. The number of cells in the image was calculated from the number of unique values above zero. The segmentation mask was then imported into the OMERO database to allow manual evaluation of the segmentation, including the identification of all single cells with no touching neighbor cells.

Co-localization (Figures 5 and 6) was calculated in both an intensity-based and an object-based process. For intensity based co-localization, images were analyzed using ImageJ to calculate Pearson's correlation coefficients applying the Costes thresholding method. For object based co-localization, puncta were automatically detected and centroided using adapted single-molecule localization routines running in Matlab. Reciprocal nearest-neighbor analyses were performed in Matlab, and the distances between assigned objects were calculated. The resulting distance distributions were thresholded based on the calculated FWHM of the microscope point spread function to identify those puncta that essentially show unresolvable co-localization.

SUPPLEMENTAL INFORMATION

Supplemental Information includes two figures, one table, and three movies and can be found with this article online at http://dx.doi.org/10.1016/j.celrep.2015.08.053.

AUTHOR CONTRIBUTIONS

Study conception and experimental design: A.J.G., A.D., M.P., C.R., J.P.A., N.R.S.-W., and S.J.C.; experimental work and data analysis: A.J.G., A.D., K.T., M.P., C.R., J.P.A., N.R.S.-W., and S.J.C.; manuscript preparation: A.D., C.R., J.P.A., N.R.S.-W., and S.J.C.

ACKNOWLEDGMENTS

Financial support was provided by the Wellcome Trust (PhD studentship to A.J.G. and personal fellowship 104556/Z/14/Z to S.J.C.), the Royal Society of Edinburgh (personal fellowship to S.J.C.), EMBO (ALTF 170-2011) and the John Fell Fund (personal fellowships to A.D.), and the MRC (grants MR/K000111X/1 and MR/K018639/1). Imaging and image analysis was supported by two Wellcome Trust Strategic Awards (097945/B/11/Z and 095931/Z/11/Z). The authors thank Dr. Sam Swift and the Dundee Imaging Facility for help with microscopy, Prof. Jason Swedlow for support and discussion, and Prof. Frank Sargent for the gift of anti-mCherry antibody.

Received: May 21, 2015
Revised: July 24, 2015
Accepted: August 18, 2015
Published: September 17, 2015

REFERENCES

Allan, C., Burel, J.M., Moore, J., Blackburn, C., Linkert, M., Loynton, S., Macdonald, D., Moore, W.J., Neves, C., Patterson, A., et al. (2012). OMERO: flexible, model-driven data management for experimental biology. Nat. Methods *9*, 245–253.

Basler, M., and Mekalanos, J.J. (2012). Type 6 secretion dynamics within and between bacterial cells. Science *337*, 815.

Basler, M., Pilhofer, M., Henderson, G.P., Jensen, G.J., and Mekalanos, J.J. (2012). Type VI secretion requires a dynamic contractile phage tail-like structure. Nature *483*, 182–186.

Basler, M., Ho, B.T., and Mekalanos, J.J. (2013). Tit-for-tat: type VI secretion system counterattack during bacterial cell-cell interactions. Cell *152*, 884–894.

Borgeaud, S., Metzger, L.C., Scrignari, T., and Blokesch, M. (2015). The type VI secretion system of Vibrio cholerae fosters horizontal gene transfer. Science *347*, 63–67.

Brunet, Y.R., Espinosa, L., Harchouni, S., Mignot, T., and Cascales, E. (2013). Imaging type VI secretion-mediated bacterial killing. Cell Rep. *3*, 36–41.

Brunet, Y.R., Hénin, J., Celia, H., and Cascales, E. (2014). Type VI secretion and bacteriophage tail tubes share a common assembly pathway. EMBO Rep. *15*, 315–321.

Casabona, M.G., Silverman, J.M., Sall, K.M., Boyer, F., Couté, Y., Poirel, J., Grunwald, D., Mougous, J.D., Elsen, S., and Attree, I. (2013). An ABC transporter and an outer membrane lipoprotein participate in posttranslational activation of type VI secretion in *Pseudomonas aeruginosa*. Environ. Microbiol. *15*, 471–486.

Coulthurst, S.J., Williamson, N.R., Harris, A.K., Spring, D.R., and Salmond, G.P. (2006). Metabolic and regulatory engineering of *Serratia marcescens*: mimicking phage-mediated horizontal acquisition of antibiotic biosynthesis and quorum-sensing capacities. Microbiology *152*, 1899–1911.

Dong, T.G., Ho, B.T., Yoder-Himes, D.R., and Mekalanos, J.J. (2013). Identification of T6SS-dependent effector and immunity proteins by Tn-seq in *Vibrio cholerae*. Proc. Natl. Acad. Sci. USA *110*, 2623–2628.

Durand, E., Cambillau, C., Cascales, E., and Journet, L. (2014). VgrG, Tae, Tle, and beyond: the versatile arsenal of Type VI secretion effectors. Trends Microbiol. *22*, 498–507.

English, G., Trunk, K., Rao, V.A., Srikannathasan, V., Hunter, W.N., and Coulthurst, S.J. (2012). New secreted toxins and immunity proteins encoded within the type VI secretion system gene cluster of *Serratia marcescens*. Mol. Microbiol. *86*, 921–936.

Felisberto-Rodrigues, C., Durand, E., Aschtgen, M.S., Blangy, S., Ortiz-Lombardia, M., Douzi, B., Cambillau, C., and Cascales, E. (2011). Towards a structural comprehension of bacterial type VI secretion systems: characterization of the TssJ-TssM complex of an *Escherichia coli* pathovar. PLoS Pathog. *7*, e1002386.

Förster, A., Planamente, S., Manoli, E., Lossi, N.S., Freemont, P.S., and Filloux, A. (2014). Coevolution of the ATPase ClpV, the sheath proteins TssB and TssC, and the accessory protein TagJ/HsiE1 distinguishes type VI secretion classes. J. Biol. Chem. *289*, 33032–33043.

Fritsch, M.J., Trunk, K., Diniz, J.A., Guo, M., Trost, M., and Coulthurst, S.J. (2013). Proteomic identification of novel secreted antibacterial toxins of the *Serratia marcescens* type VI secretion system. Mol. Cell. Proteomics *12*, 2735–2749.

Ho, B.T., Dong, T.G., and Mekalanos, J.J. (2014). A view to a kill: the bacterial type VI secretion system. Cell Host Microbe *15*, 9–21.

Iguchi, A., Nagaya, Y., Pradel, E., Ooka, T., Ogura, Y., Katsura, K., Kurokawa, K., Oshima, K., Hattori, M., Parkhill, J., et al. (2014). Genome evolution and plasticity of *Serratia marcescens*, an important multidrug-resistant nosocomial pathogen. Genome Biol. Evol. *6*, 2096–2110.

Kaniga, K., Delor, I., and Cornelis, G.R. (1991). A wide-host-range suicide vector for improving reverse genetics in gram-negative bacteria: inactivation of the *blaA* gene of *Yersinia enterocolitica*. Gene *109*, 137–141.

Kapitein, N., Bönemann, G., Pietrosiuk, A., Seyffer, F., Hausser, I., Locker, J.K., and Mogk, A. (2013). ClpV recycles VipA/VipB tubules and prevents non-productive tubule formation to ensure efficient type VI protein secretion. Mol. Microbiol. *87*, 1013–1028.

Kube, S., Kapitein, N., Zimniak, T., Herzog, F., Mogk, A., and Wendler, P. (2014). Structure of the VipA/B type VI secretion complex suggests a contraction-state-specific recycling mechanism. Cell Rep. *8*, 20–30.

Kuhn, A. (2014). Introduction to special issue on protein trafficking and secretion. Biochim. Biophys. Acta *1843*, 1429–1432.

Leake, M.C., Chandler, J.H., Wadhams, G.H., Bai, F., Berry, R.M., and Armitage, J.P. (2006). Stoichiometry and turnover in single, functioning membrane protein complexes. Nature *443*, 355–358.

LeRoux, M., De Leon, J.A., Kuwada, N.J., Russell, A.B., Pinto-Santini, D., Hood, R.D., Agnello, D.M., Robertson, S.M., Wiggins, P.A., and Mougous, J.D. (2012). Quantitative single-cell characterization of bacterial interactions reveals type VI secretion is a double-edged sword. Proc. Natl. Acad. Sci. USA *109*, 19804–19809.

Lybarger, S.R., Johnson, T.L., Gray, M.D., Sikora, A.E., and Sandkvist, M. (2009). Docking and assembly of the type II secretion complex of *Vibrio cholerae*. J. Bacteriol. *191*, 3149–3161.

Ma, L.S., Lin, J.S., and Lai, E.M. (2009). An IcmF family protein, ImpLM, is an integral inner membrane protein interacting with ImpKL, and its walker a motif is required for type VI secretion system-mediated Hcp secretion in *Agrobacterium tumefaciens*. J. Bacteriol. *191*, 4316–4329.

Mahlen, S.D. (2011). *Serratia* infections: from military experiments to current practice. Clin. Microbiol. Rev. *24*, 755–791.

Mougous, J.D., Gifford, C.A., Ramsdell, T.L., and Mekalanos, J.J. (2007). Threonine phosphorylation post-translationally regulates protein secretion in *Pseudomonas aeruginosa*. Nat. Cell Biol. *9*, 797–803.

Murdoch, S.L., Trunk, K., English, G., Fritsch, M.J., Pourkarimi, E., and Coulthurst, S.J. (2011). The opportunistic pathogen *Serratia marcescens* utilizes type VI secretion to target bacterial competitors. J. Bacteriol. *193*, 6057–6069.

Petersen, L.M., and Tisa, L.S. (2013). Friend or foe? A review of the mechanisms that drive *Serratia* towards diverse lifestyles. Can. J. Microbiol. *59*, 627–640.

Plank, M., Wadhams, G.H., and Leake, M.C. (2009). Millisecond timescale slimfield imaging and automated quantification of single fluorescent protein molecules for use in probing complex biological processes. Integr. Biol. (Camb). *1*, 602–612.

Russell, A.B., Peterson, S.B., and Mougous, J.D. (2014). Type VI secretion system effectors: poisons with a purpose. Nat. Rev. Microbiol. *12*, 137–148.

Schwarz, S., Singh, P., Robertson, J.D., LeRoux, M., Skerrett, S.J., Goodlett, D.R., West, T.E., and Mougous, J.D. (2014). VgrG-5 is a *Burkholderia* type VI secretion system-exported protein required for multinucleated giant cell formation and virulence. Infect. Immun. *82*, 1445–1452.

Shneider, M.M., Buth, S.A., Ho, B.T., Basler, M., Mekalanos, J.J., and Leiman, P.G. (2013). PAAR-repeat proteins sharpen and diversify the type VI secretion system spike. Nature *500*, 350–353.

Silverman, J.M., Agnello, D.M., Zheng, H., Andrews, B.T., Li, M., Catalano, C.E., Gonen, T., and Mougous, J.D. (2013). Haemolysin coregulated protein is an exported receptor and chaperone of type VI secretion substrates. Mol. Cell *51*, 584–593.

Srikannathasan, V., English, G., Bui, N.K., Trunk, K., O'Rourke, P.E.F., Rao, V.A., Vollmer, W., Coulthurst, S.J., and Hunter, W.N. (2013). Structural basis for type VI secreted peptidoglycan DL-endopeptidase function, specificity and neutralization in *Serratia marcescens*. Acta Crystallogr. D Biol. Crystallogr. *69*, 2468–2482.

Stanley, N.R., Britton, R.A., Grossman, A.D., and Lazzazera, B.A. (2003). Identification of catabolite repression as a physiological regulator of biofilm formation by *Bacillus subtilis* by use of DNA microarrays. J. Bacteriol. *185*, 1951–1957.

Whitney, J.C., Beck, C.M., Goo, Y.A., Russell, A.B., Harding, B.N., De Leon, J.A., Cunningham, D.A., Tran, B.Q., Low, D.A., Goodlett, D.R., et al. (2014). Genetically distinct pathways guide effector export through the type VI secretion system. Mol. Microbiol. *92*, 529–542.

Zoued, A., Brunet, Y.R., Durand, E., Aschtgen, M.S., Logger, L., Douzi, B., Journet, L., Cambillau, C., and Cascales, E. (2014). Architecture and assembly of the Type VI secretion system. Biochim. Biophys. Acta *1843*, 1664–1673.

Cell Reports

Supplemental Information

Visualization of the *Serratia* Type VI Secretion System Reveals Unprovoked Attacks and Dynamic Assembly

Amy J. Gerc, Andreas Diepold, Katharina Trunk, Michael Porter, Colin Rickman, Judith P. Armitage, Nicola R. Stanley-Wall, and Sarah J. Coulthurst

Figure S1

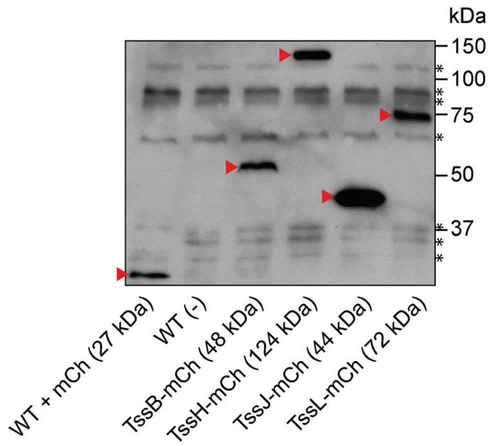

Figure S1. Confirmation of the integrity of chromosomally-encoded fusion proteins of Type VI secretion system components with mCherry, related to Figure 1.

Total cellular fractions of wild type *Serratia marcescens* Db10 (WT) and derivatives expressing fusions of mCherry to the C-terminus of TssB (TssB-mCh), TssH (TssH-mCh), TssJ (TssJ-mCh) or TssL (TssL-mCh) were subjected to immunoblotting using anti-mCherry antisera. A positive control is provided by the WT expressing mCherry from a plasmid (WT + mCh). The predicted molecular weight of each protein is given in parentheses. The mCherry fusion proteins are highlighted with red arrowheads and the positions of non-specific bands are indicated by asterisks on the right hand side.

Figure S2

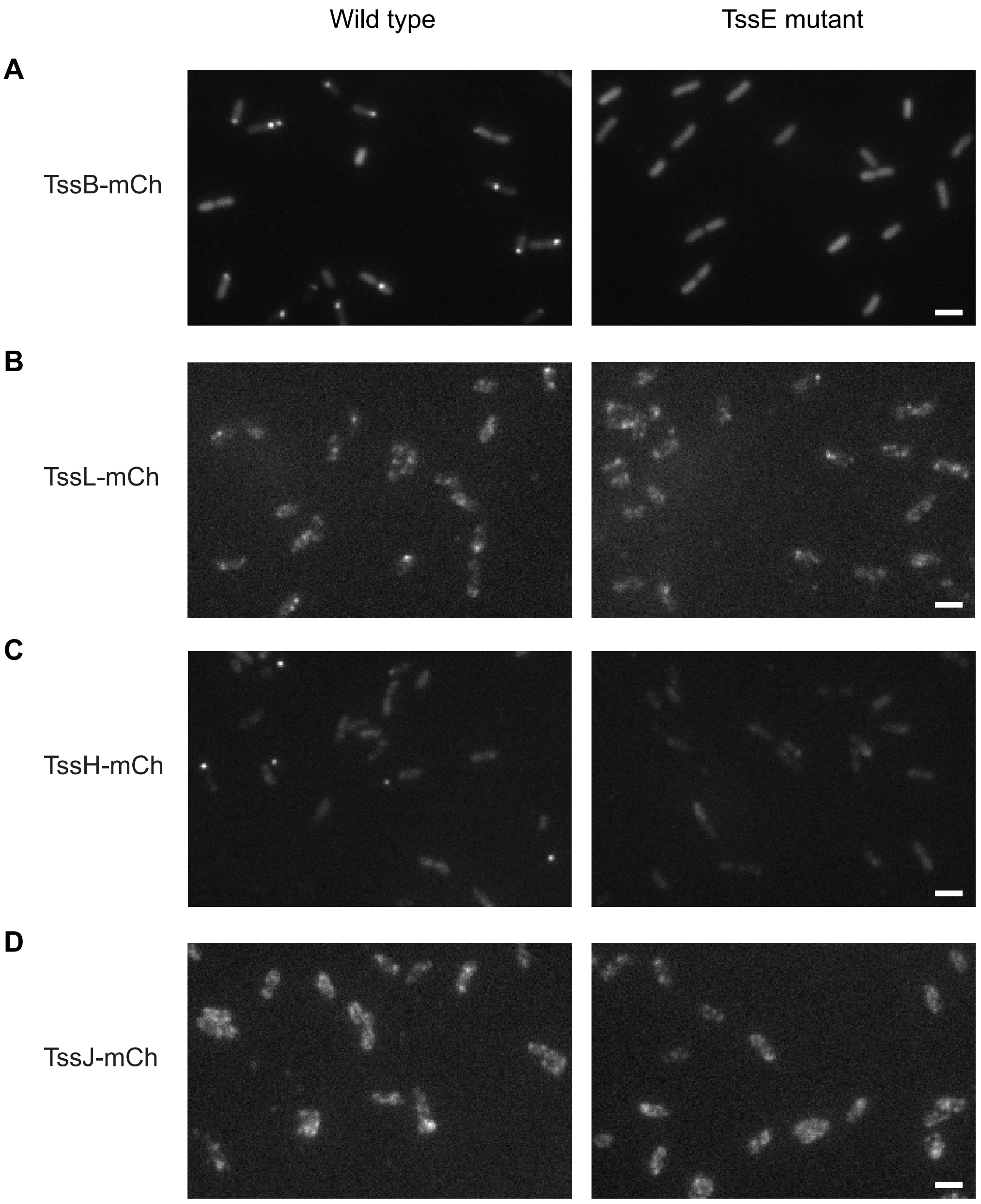

Figure S2. Localisation of TssB, TssH, TssJ and TssL in the absence of the essential baseplate protein TssE, related to Figure 4.
Representative fluorescence images of cells of *Serratia marcescens* Db10 expressing chromosomally-encoded fusion proteins TssB-mCh (A), TssL-mCh (B), TssH-mCh (C) or TssJ-mCh (D), in either a wild type (left) or Δ*tssE* mutant (right) background; scale bar, 2 μm.

Legends for Supplemental Movies

Movie S1. Fluorescence time course of TssB-mCherry during microcolony development, related to Figure 2.
Images were acquired at 10 min intervals for a total period of 6 hours, for the field of view containing the cells shown in Fig. 2. Left panel, DIC images; middle panel, corresponding fluorescence images (mCherry channel); right panel, false-coloured merge of DIC images (greyscale) and fluorescence images (mCherry, red).

Movie S2. Fluorescence time course of TssB-mCherry, related to Figure 4E.
Images were acquired at 10 s intervals for 4 minutes.

Movie S3. Fluorescence time course of TssH-mCherry, related to Figure 4F.
Images were acquired at 10 s intervals for 4 minutes.

Table S1. Bacterial strains used in this study, related to Experimental Procedures

Strain	Description	Source or Reference
S. marcescens Db10	Wild type strain	(Flyg et al., 1980; Iguchi et al., 2014)
SJC3	Db10 Δ*tssH* (ΔSMDB11_2274)	(Murdoch et al., 2011)
SJC11	Db10 Δ*tssE* (ΔSMDB11_2271)	(Murdoch et al., 2011)
SAN157	Db10 *tssJ-mCherry* (TssJ-mCh translational fusion; TssJ, SMDB11_2252)	This study
SAN159	Db10 *tssH-mCherry* (TssH-mCh translational fusion; TssH, SMDB11_2274)	This study
SAN162	Db10 *tssL-mCherry* (TssL-mCh translational fusion; TssL, SMDB11_2254)	This study
SAN163	Db10 *tssB-mCherry* (TssB-mCh translational fusion; TssB, SMDB11_2258)	This study
SAN199	Db10 *tssB-mCherry, ΔlacZ*::P_{T5}-*gfpmut2-kan*^{R} (TssB-mCh translational fusion and cytoplasmic GFP; LacZ, SMDB11_2462)	This study
SAN207	Db10 *tssB-gfpmut2*; TssB-GFP translational fusion	This study
SAN208	Db10 *tssB-gfpmut2, tssJ-mCherry* (dual reporter fusion strain)	This study
SAN209	Db10 *tssB-gfpmut2, tssH-mCherry* (dual reporter fusion strain)	This study
SAN210	Db10 *tssB-gfpmut2, tssL-mCherry* (dual reporter fusion strain)	This study
KT24	Db10 ΔT6SS (ΔSMDB11_2244-SMDB11_2281), Sm-resistant	(English et al., 2012)
KT153	Db10 *tssJ-mCherry*, Δ*tssE*	This study
KT154	Db10 *tssH-mCherry*, Δ*tssE*	This study
KT155	Db10 *tssL-mCherry*, Δ*tssE*	This study
KT156	Db10 *tssB-mCherry*, , Δ*tssE*	This study
S. marcescens ATCC274	Wild type strain	Lab stock
SJC44	ATCC274 Δ*tssE*	This study
KT81	Sm-resistant derivative of ATCC274	This study
KT82	Sm-resistant derivative of SJC44	This study
P. fluorescens KT02	Sm-resistant derivative of *Pseudomonas fluorescens* 55	(Murdoch et al., 2011)

Supplemental Reference

Flyg, C., Kenne, K., and Boman, H.G. (1980). Insect pathogenic properties of *Serratia marcescens*: phage-resistant mutants with a decreased resistance to *Cecropia* immunity and a decreased virulence to *Drosophila*. J Gen Microbiol *120*, 173-181.
